# Secretory expression of recombinant small laccase genes in Gram-positive bacteria

**DOI:** 10.1186/s12934-023-02075-5

**Published:** 2023-04-17

**Authors:** Silja Välimets, Patricia Pedetti, Ludovika Jessica Virginia, Mai Ngoc Hoang, Michael Sauer, Clemens Peterbauer

**Affiliations:** 1 Department of Food Sciences and Technology, University of Natural Resources and Life Sciences, Institute of Food Technology, Muthgasse 18, Vienna, Vienna, 1190 Austria; 2 Department of Biotechnology, Institute of Microbiology and Microbial Biotechnology, University of Natural Resources and Life Sciences, Muthgasse 18, Vienna, Vienna, 1190 Austria; 3 Food Microbiology, Wageningen University and Research, Droevendaalsesteeg 4, Wageningen, 6708 PB The Netherlands; 4 Department of Human Medicine, Institute of Immunology, Carl von Ossietzky University of Oldenburg, Carl-von-Ossietzky-Straße 9-11, 26129 Oldenburg, Germany

**Keywords:** *Streptomyces lividans*, *Bacillus subtilis*, Heterologous expression, Bacterial protein secretion

## Abstract

**Background:**

Laccases are multicopper enzymes that oxidize a wide range of aromatic and non-aromatic compounds in the presence of oxygen. The majority of industrially relevant laccases are derived from fungi and are produced in eukaryotic expression systems such as *Pichia pastoris* and *Saccharomyces cerevisiae*. Bacterial laccases for research purposes are mostly produced intracellularly in *Escherichia coli*, but secretory expression systems are needed for future applications. Bacterial laccases from *Streptomyces* spp. are of interest for potential industrial applications because of their lignin degrading activities.

**Results:**

In this study, we expressed small laccases genes from *Streptomyces coelicolor*, *Streptomyces viridosporus* and *Amycolatopsis* 75iv2 with their native signal sequences in Gram-positive *Bacillus subtilis* and *Streptomyces lividans* host organisms. The extracellular activities of *Sc*Lac, *Sv*Lac and *Am*Lac expressed in *S. lividans* reached 1950 ± 99 U/l, 812 ± 57 U/l and 12 ± 1 U/l in the presence of copper supplementation. The secretion of the small laccases was irrespective of the copper supplementation; however, activities upon reconstitution with copper after expression were significantly lower, indicating the importance of copper during laccase production. The production of small laccases in *B. subtilis* resulted in extracellular activity that was significantly lower than in *S. lividans*. Unexpectedly, *Am*Lac and *Sc*Lac were secreted without their native signal sequences in *B. subtilis*, indicating that *B. subtilis* secretes some heterologous proteins via an unknown pathway.

**Conclusions:**

Small laccases from *S. coelicolor*, *S. viridosporus* and *Amycolatopsis* 75iv2 were secreted in both Gram-positive expression hosts *B. subtilis* and *S. lividans*, but the extracellular activities were significantly higher in the latter.

**Supplementary Information:**

The online version contains supplementary material available at 10.1186/s12934-023-02075-5.

## Introduction

Laccase activity was first described in 1883 [1]. Laccases are multicopper enzymes that oxidize aromatic and non-aromatic substrates while reducing oxygen to water. The four copper (II) ions in the active site are divided into type one (T1), type two (T2) and two type three (T3) coppers which are distinguished by their spectroscopic features and roles in the catalytic cycle. Substrate is oxidized in the proximity of T1, whereas reduction of oxygen to water happens near the T2 and T3 cluster [2].

Laccases are distributed among bacteria, insects, plants and fungi [3]. The physiological role of laccases varies significantly in these organisms. Fungal laccases protect against stress and participate in lignin degradation; plant laccases are involved in wound response and lignin polymerization; bacterial laccases oxidize toxins, protect against UV light, participate in lignin degradation and play a role in spore formation [3, 4]. The versatility of laccases has led to new applications in biotechnology, for example removal of polyphenols in wine and beer industry [5], treatment of textiles for the clothing industry [6, 7], applications in the pulp and paper industry [8], removal of phenolic components for increased bioethanol production [9], and bioremediation [10–13].

The largest number of laccases for industrial applications are from fungi because of extensive research and higher redox potential compared to bacterial laccases [14, 15]. Industrial enzymes are usually produced as secreted enzymes by a suitable host system, if the enzyme in question is naturally secreted. Industrially used fungal laccases are mainly produced in *Saccharomyces cerevisiae, Pichia pastoris, Trichoderma reesei, Aspergillus niger* or others [16].

A number of laccases from *Streptomyces* spp. and *Bacillus* spp. related species have been found in the secretomes of their natural hosts [17, 18]. The laccases from *Streptomyces* spp. have been termed small laccases because of the smaller size of the enzyme compared to the fungal laccases [19]. The small laccases from *Amycolatopsis* 75iv2 (*Am*Lac), *Streptomyces coelicolor* (*Sc*Lac) and *Streptomyces viridosporus* (*Sv*Lac) have been shown to play a role in lignin degradation and modification [20] and may be promising candidates for biocatalytic applications. Lignin is a heterogeneous aromatic polymer that cannot be taken up by the cells and is generally depolymerized by unspecific oxidative secretory enzymes in the extracellular space [21].

Heterologous expression of bacterial laccases genes is often performed in *E. coli* [12, 19, 20, 22–24]. However, intracellular protein production in *E. coli* requires additional downstream processes or may even result in an inactive enzyme [25].

The Gram-positive bacterium *B. subtilis* is well established as an industrial expression host for a number of bacterial secretory proteins (homologous and heterologous) [26, 27]. The advantages of *B. subtilis* as an expression host are (1) non-toxicity due to the absence of lipopolysaccharides (LPS) (2) available molecular tools such as shuttle vectors, signal peptide libraries [28, 29], and promoter systems [30, 31] (3) fully sequenced genome since 1997 [32] (4) fast growth rate with an average doubling time as low as 2 h [33] (5) cultivation in cheap media and robustness in cultivation and (6) secretion capacity. Examples of heterologously produced and secreted proteins with high titers are α-amylase [34] and human epidermal growth factor [35]. The major disadvantage of *B. subtilis* as an expression host is the secretion of endogenous proteases that can degrade produced secretory proteins. The *B. subtilis* genome encodes at least 7 proteases including alkaline protease (aprE), neutral proteases (nprE and nprB), metalloprotease (mpr) and three serine proteases (epr, bpf, vpr). To tackle this problem, several protease-deficient strains have been constructed and applied for protein production [36].

Another Gram-positive bacterium suitable for heterologous production and secretion is the aerobic soil bacterium *Streptomyces lividans* that is mainly recognized as an antibiotic producer [37]. The advantages of the *S. lividans* expression system are non-toxicity due to the absence of LPS and low extracellular proteolytic activity [38]. As a GC-rich bacterium, it is well suited for the expression of genes from other GC-rich microorganisms [39]. Some examples of heterologously produced and secreted enzymes in *S. lividans* are endoglucanase [40], phospholipase D [41], and cellulase [42]. The disadvantages of *S. lividans* are (1) G- and C-nucleotide rich genome, which could result in biased codon usage [43] and unsuccessful expression of codon optimized genes (2) no commercially available molecular tools (3) mycelial growth resulting in heterogeneous culture and (4) long doubling time of 6 h [44].

The two main secretion systems in Gram-positive bacteria are the Sec and the Tat (twin-arginine translocation) pathways. Proteins are secreted in an unfolded state via the Sec pathway (and are folded into their functional conformation immediately after secretion), whereas the Tat pathway is used for the translocation of functionally folded proteins (often enzymes with complex cofactors) [45, 46]. The general requirement for translocation of an enzyme is an N-terminal signal sequence. The signal sequences vary in length, but they have typical structural features such as a positively charged N-terminus (N region), hydrophobic residues in the central part (H region) and more polar C terminus [47]. Based on this, several prediction tools have been developed for example SignalP [48] and Pred-TAT [49, 50].

In this study, we explore the heterologous secretory expressionof small laccases genes from *S. coelicolor*, *S. viridosporus* and *Amycolatopsis* 75iv2 in *B. subtilis* and *S. lividans*. The establishment of an efficient production and secretion system for bacterial ligninolytic enzymes is an important milestone for future applications.

## Results

### Small laccases have predicted tat-pathway signal sequences

**Fig. 1:**
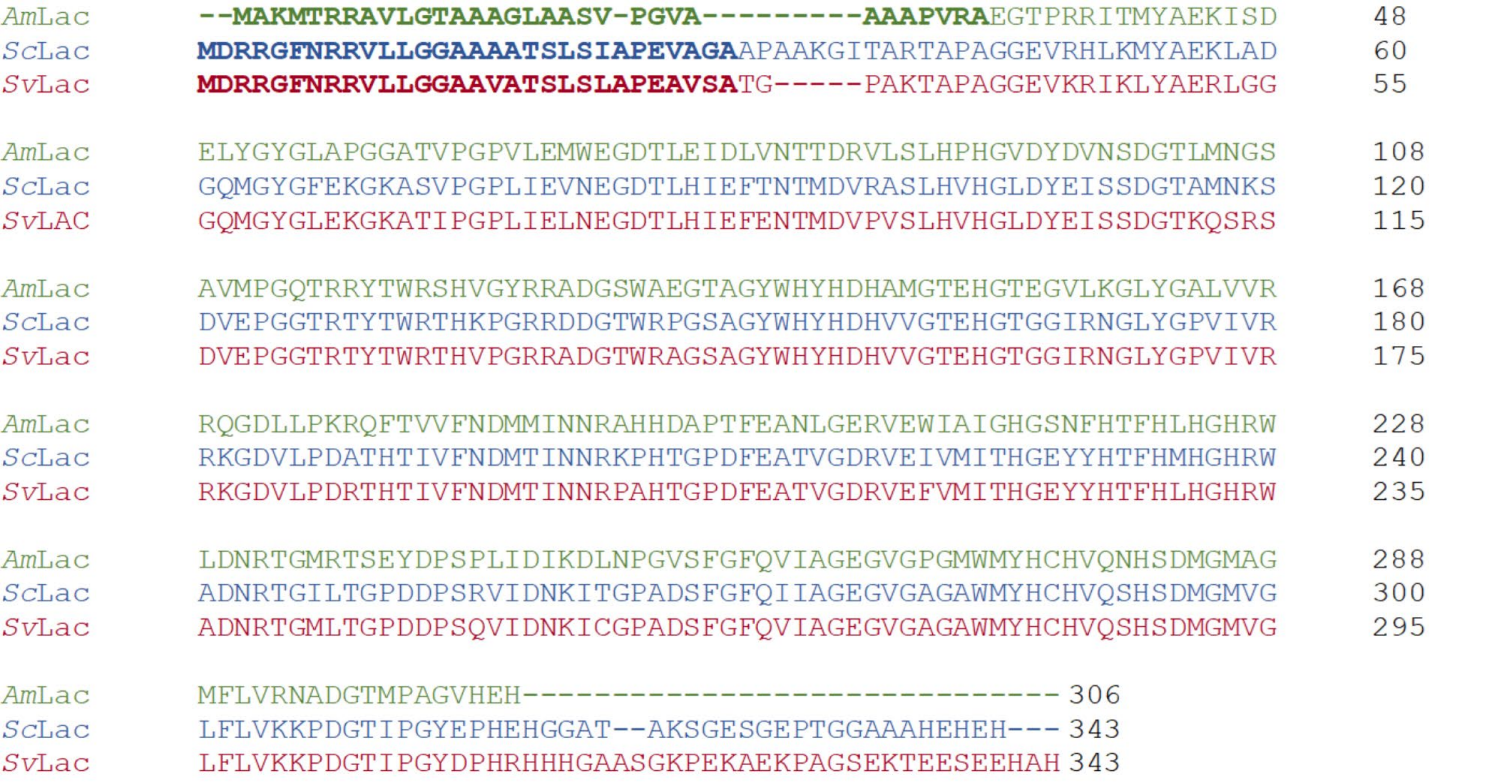
Small laccases and their predicted signal sequences used in this study. The small laccases from *Amycolatopsis* 75iv2 (*Am*Lac), *Streptomyces coelicolor* (*Sc*Lac) and *Streptomyces viridosporus* (*Sv*Lac) have predicted twin arginine translocation (tat) pathway signal sequences marked in bold. The signal sequences were predicted using SignalP 5.0 algorithm from DTU, Denmark. The sequences were aligned using Clustal Omega

The amino acid sequences of the small laccases were analyzed with SignalP 5.0. The predicted signal sequences are shown in Fig. 1 and in Supplementary Fig. 1. *Am*Lac, *Sc*Lac, *Sv*Lac signal sequences were 32, 30 and 30 amino acids long, respectively. The predicted signal sequence for *Am*Lac, *Sc*Lac and *Sv*Lac are predicted to be cleaved between VRA and EG, AGA and AP, VSA and TG amino acids, respectively (Supplementary Fig. 1). With the likelihood of greater than 0.9, all the small laccases have a twin-arginine translocation (Tat) pathway signal sequence (Supplementary Fig. 1). These results suggest that the small laccases are naturally secreted.

### Heterologous expression of recombinant small laccases in ***S. lividans***

**Fig. 2:**
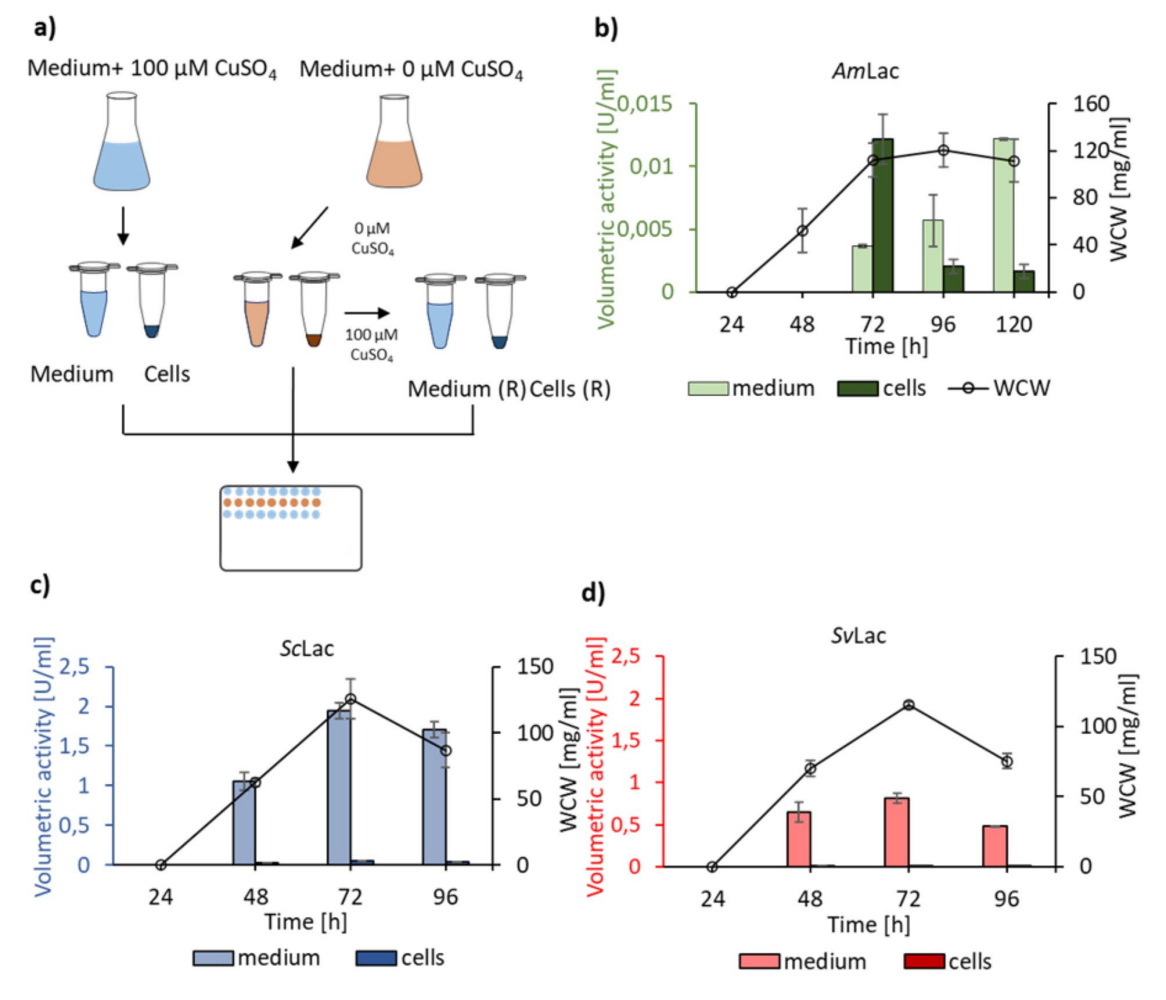
The expression of small laccases genes in *S. lividans* host organism. (a) Experimental set up for the small laccases production and secretion studies. Bacteria were cultivated with the growth medium containing 0 or 100 µM of CuSO_4_. Samples at defined time points were harvested and the samples without CuSO_4_ were divided into two aliquots. The first aliquot was saved for control measurements. The second aliquot was reconstituted by supplementing with 100 µM CuSO_4_, incubated overnight and measured on the next day. Reconstituted samples were named medium (R) and cells (R). **(b)** Secretion of *Am*Lac, **(c)** secretion of *Sc*Lac and **(d)** secretion of *Sv*Lac. The spores were cultivated in the growth medium containing 100 µM of CuSO_4_. The samples were taken at defined time points, medium and cells were separated by centrifugation and activity was measured spectrophotometrically. The wet cell weight (WCW) was measured to follow bacterial growth. After 24 h no cell growth was observed. The activity measurements are an average of three biological replicates in three technical replicates each

Small laccases were produced in the phylogenetically closely related host organism *S. lividans* TK24. To ensure fully active enzymes with a full complement of copper in the active site, the growth medium was supplemented with 100 µM CuSO_4_ (Fig. 2a). The activity of the small laccases was measured using ABTS as a substrate. After 24 h minimal cell growth was observed (Supplementary Fig. 2). Extracellular activity of *Sc*Lac, *Sv*Lac was detected after 48 h (Fig. 2c and d) and reached a maximum after 72 h. The increase in activity correlated to the increase of wet cell weight. The intracellular fraction (cells) only presented residual activity indicating efficient secretion (Fig. 2c and d). *S. lividans* expressing *Am*Lac showed different growth and volumetric activity compared to *Sc*Lac and *Sv*Lac. The strain expressing *Am*Lac (Fig. 2b) formed fewer spores, stayed longer in the stationary phase and presented significantly lower volumetric activity than *Sc*Lac or *Sv*Lac.

**Fig. 3:**
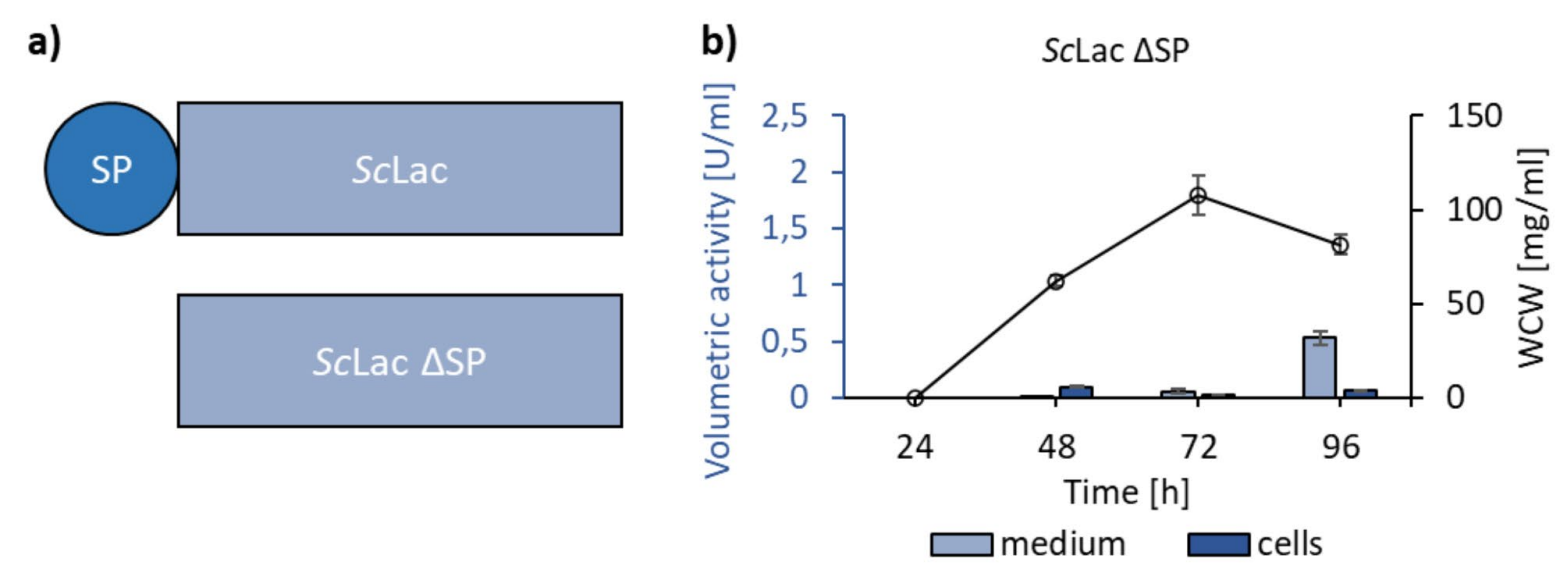
The expression of *Sc* Lac without signal sequence. **(a)** Schematic presentation of *Sc*Lac gene with native signal sequence and *Sc*Lac ∆SP without native signal sequence. **(b)** Secretion of *Sc*Lac∆SP. The spores were cultivated in the growth media containing 100 µM of CuSO_4_. The samples were taken at defined time points, medium and cells were separated by centrifugation and activity was measured spectrophotometrically. The wet cell weight (WCW) was measured to follow bacterial growth. After 24 h no cell growth was observed. The activity measurements are the average of three biological replicates in three technical replicates each

To verify that the predicted signal sequences are functionally translocating the enzymes to the extracellular space and to rule out that the extracellular activity was caused by cell lysis, *Sc*Lac and *Sv*Lac were expressed without their native signal sequences as shown in Fig. 1 (marked bold) and Fig. 3a. No extracellular activity of *Sc*Lac ∆SP was detected after 48 and 72 h. A significant increase in the extracellular activity of *Sc*Lac ∆SP was only detected after 96 h indicating cell lysis (Fig. 3b). Thus, the observed activity of *Sc*Lac with the signal sequence in 48 and 72 h was not due to cell lysis. *Sv*Lac ∆SP did not present extracellular or intracellular activity. Finally, to verify that the enzyme was processed during the translocation process, the secreted *Sc*Lac was subjected to mass-spectrometry analysis (Supplementary Fig. 3). No peptides corresponding to the signal sequence were detected indicating that the enzyme was correctly processed.

### The effect of copper ions on the recombinant small laccase secretion in *S. lividans*

**Fig. 4:**
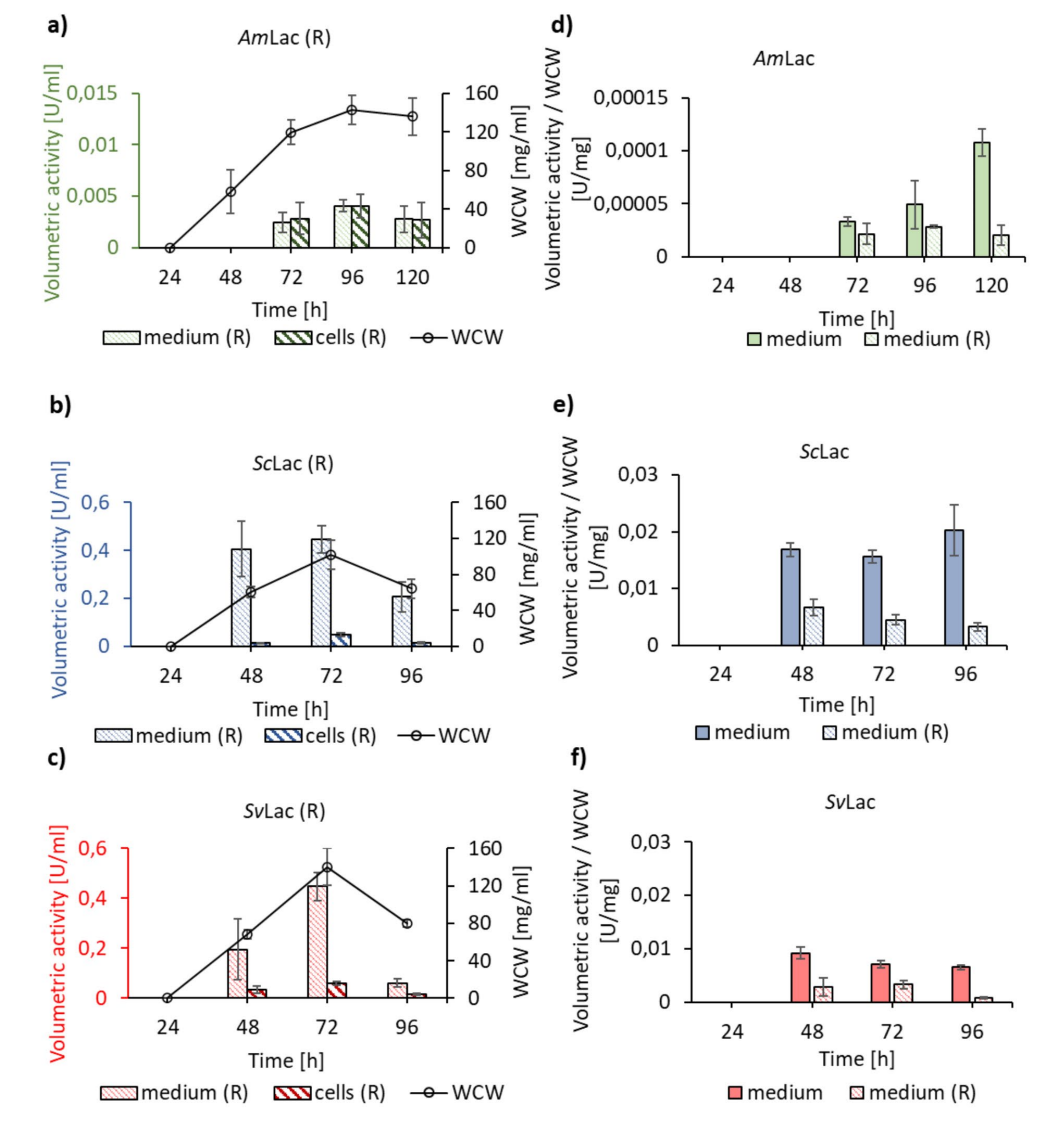
The effect of copper in the secretion of the small laccases. Secretion of **a) ***Am*Lac, **b) ***Sc*Lac, and **c) ***Sv*Lac in the absence of additional copper supplementation. Spores were inoculated into growth media containing 0 µM of CuSO_4_. Samples were taken at defined time points and the cells were separated by centrifugation. The supernatants were aliquoted into two separate samples, one was saved as a control. The second aliquot was reconstituted by supplementing with 100 µM of CuSO_4_, incubated overnight and measured on the next day. The reconstituted samples were named medium (R) and cells (R). The reconstituted activities of *Am*Lac, *Sc*Lac and *Sv*Lac were detected in the medium indicating that secretion was happening independently of copper. The activity results were the average of three biological replicates in three technical replicates each. The comparison of **d) ***Am*Lac, **e) ***Sc*Lac, or **f) ***Sv*Lac volumetric activities in the absence and presence of copper. The volumetric activities were normalized to WCW. Higher activity was measured in the medium when copper was present at the beginning of the expression

Laccases require copper ions in the active site to oxidize a variety of compounds. To study the role of copper ions in the secretion of the small laccases, protein production was conducted without additional copper supplementation (Fig. 2a). At each time point samples were harvested, the supernatant was separated from the cells by centrifugation, supplemented with copper ions and incubated overnight. The enzyme activity of *Am*Lac, *Sc*Lac, and *Sv*Lac was reconstituted after copper supplementation (Fig. 4a, b and c). No laccase activity was measured before copper reconstitution. Naturally available copper from growth medium components was not enough to result in fully active small laccases. Notably, the activity of the reconstituted samples [medium (R) and cells (R)] did not reach the same levels as the non-reconstituted (medium and cells) (Fig. 4d, e and f).

### Heterologous expression of the recombinant small laccases in *B. subtilis*

Initially, we studied the production of *Sc*Lac in the industrially relevant host organism *B. subtilis. Sc*Lac gene was expressed using the **Su**btilin **r**egulated **e**xpression (SURE) system in *B. subtilis* NZ8900 strain (Table 1). Extracellular activity was only detected after 72 h suggesting the release of the intracellular content due to cell lysis. However, intracellular activity of *Sc*Lac was already detected after 24 h indicating expression but no secretion (data not shown).

**Table 1:** Bacterial strains and plasmids

Strain	Genotype or characteristics	Host	Source
*B. subtilis* NZ8900	*B. subtilis* 168 strain amyE::spaRK		MoBiTec GmbH
*B. subtilis* NZ8963	ATCC6633 subtilin producing strain		MoBiTec GmbH
*B. subtilis* RIK1285	Marburg 168 derivative: trpC2, lys1, aprE Δ3, nprR2, nprE18		Takara Bio Inc.
*E. coli* JM109	Intermediate cloning host endA1 glnV44 thi-1 relA1 gyrA96 recA1 mcrB + Δ(lac-proAB) e14- [F’ traD36 proAB + lacIq lacZΔM15] hsdR17(rK-mK+)		Lab stock
*E. coli* MC1061	Intermediate cloning host F- araD139 Δara-leu)7696 Δ(lac)X74 galU galK hsdR2 mcrA mcrB1 rspL		MoBiTec GmbH
*S. lividans* TK24	[42]		KU Leuven
**Plasmids**	**Genotype or characteristics**		
pNZ8901	Subtilin regulated expression (SURE) vector P_spaSmut_, Cm^R^	*E. coli* MC1061/ *B. subtilis* NZ8900	MoBiTec GmbH
pNZ-*Bs* SP *Am*DyP	pNZ containing signal peptide (SP) from *B. subtilis* NZ8900 dye-decolorizing peroxidase (DyP), DyP gene from *Amycolatopsis* 75iv2 (*Am*DyP), Tobacco Etch Virus cleavage site (TEV), 6 Histidine tag (6His)	*E. coli* MC1061 / *B. subtilis* NZ8900	This study
pNZ-*Am*DyP	pNZ containing gene of interest *Am*DyP, TEV, 6His	*E. coli* MC1061 / *B. subtilis* NZ8900	This study
pNZ-*Sc*Lac	pNZ containing gene of interest small laccase (Slac) from *Streptomyces coelicolor* (*Sc*Lac), TEV, 6His	*E. coli* MC1061 / *B. subtilis* NZ8900	This study
pNZ-*Sc*Lac ∆SP	pNZ containing gene of interest *Sc*Lac with the deletion of the SP, TEV, 6His	*E. coli* MC1061 / *B. subtilis* NZ8900	This study
pBE-S	pUB ori, Kan^R^, ColE1 ori, Amp^R^, aprE promoter, aprE SP, His tag	*E. coli* JM109 / *B. subtilis* RIK1285	Takara Bio Inc.
pBE-*Bs* SP *Am*DyP	pBE containing *B. subtilis* SP, *Am*DyP, TEV, 6His	*E. coli* JM109 / *B. subtilis* RIK1285	This study
pBE-*Am*DyP	pBE containing gene of interest *Am*DyP, TEV, 6His	*E. coli* JM109 / *B. subtilis* RIK1285	This study
pBE-*Am*Lac	pBE containing gene of interest Slac from *Amycolatopsis* 75iv2 (*Am*Lac), TEV, 6His	*E. coli* JM109 / *B. subtilis* RIK1285	This study
pBE-*Am*Lac ∆SP	pBE containing gene of interest *Am*Lac with the deletion of the SP, TEV, 6His	*E. coli* JM109 / *B. subtilis* RIK1285	This study
pBE-*Sc*Lac	pBE containing gene of interest *Sc*Lac, TEV, 6His	*E. coli* JM109 / *B. subtilis* RIK1285	This study
pBE- *Sc*Lac ∆SP	pBE containing gene of interest *Sc*Lac with the deletion of the SP, TEV, 6His	*E. coli* JM109 / *B. subtilis* RIK1285	This study
pBE-*Sv*Lac	pBE containing gene of interest SLAC from *Streptomyces viridosporus* (*Sv*Lac), TEV, 6His	*E. coli* JM109 / *B. subtilis* RIK1285	This study
pBE- *Sv*Lac ∆SP	pBE containing gene of interest *Sv*Lac with the deletion of the SP, TEV, 6His	*E. coli* JM109 / *B. subtilis* RIK1285	This study
pBE- *Sc*Lac ∆SP-TEV-*Am*DyP	pBE containing *Sc*Lac with the deletion of the SP, TEV, *Am*DyP, TEV, 6His	*E. coli* JM109 / *B. subtilis* RIK1285	This study
pBE- *Sc*Lac ∆SP-TEV-GFP	pBE containing *Sc*Lac with the deletion of the SP, TEV, GFP, TEV, 6His	*E. coli* JM109 / *B. subtilis* RIK1285	This study
pIJ486	P_vsi_, Rep ori, Ts^R^, Kan^R^	*S. lividans* TK24	KU Leuven
pIJ- *Sc*Lac	pIJ containing *Sc*Lac, TEV, 6His	*S. lividans* TK24	This study
pIJ- *Sc*Lac ∆SP	pIJ containing *Sc*Lac with the deletion of the SP, TEV, 6His	*S. lividans* TK24	This study
pIJ- *Am*Lac	pIJ containing *Am*Lac, TEV, 6His	*S. lividans* TK24	This study
pIJ- *Sv*Lac	pIJ containing *Sv*Lac, TEV, 6HIs	*S. lividans* TK24	This study
pIJ-*Sc* SP-*Am*DyP	pIJ containing SP from *Sc*Lac, *Am*DyP, TEV, 6His	*S. lividans* TK24	This study
pUC19-P_vsi_	P_vsi_, Rep ori, Amp^R^, Amp^R^ promoter, CAP binding site, lac promoter, lacZα	*E. coli* JM109	[39]
pUC-*Sc*Lac	pUC containing P_vsi_, *Sc*Lac, TEV, 6His	*E. coli* JM109	This study
pUC-*Sc*Lac ∆SP	pUC containing P_vsi_, *Sc*Lac with the deletion of the SP, TEV, 6His	*E. coli* JM109	This study
pUC-*Am*Lac	pUC containing P_vsi_, *Am*Lac, TEV, 6His	*E. coli* JM109	This study
pUC-*Sv*Lac	pUC containing P_vsi_, *Sv*Lac, TEV, 6His	*E. coli* JM109	This study
pUC-*Sc* SP-*Am*DyP	pUC containing P_vsi_, SP from *Sc*Lac, *Am*DyP, TEV, 6His	*E. coli* JM109	This study

**Fig. 5:**
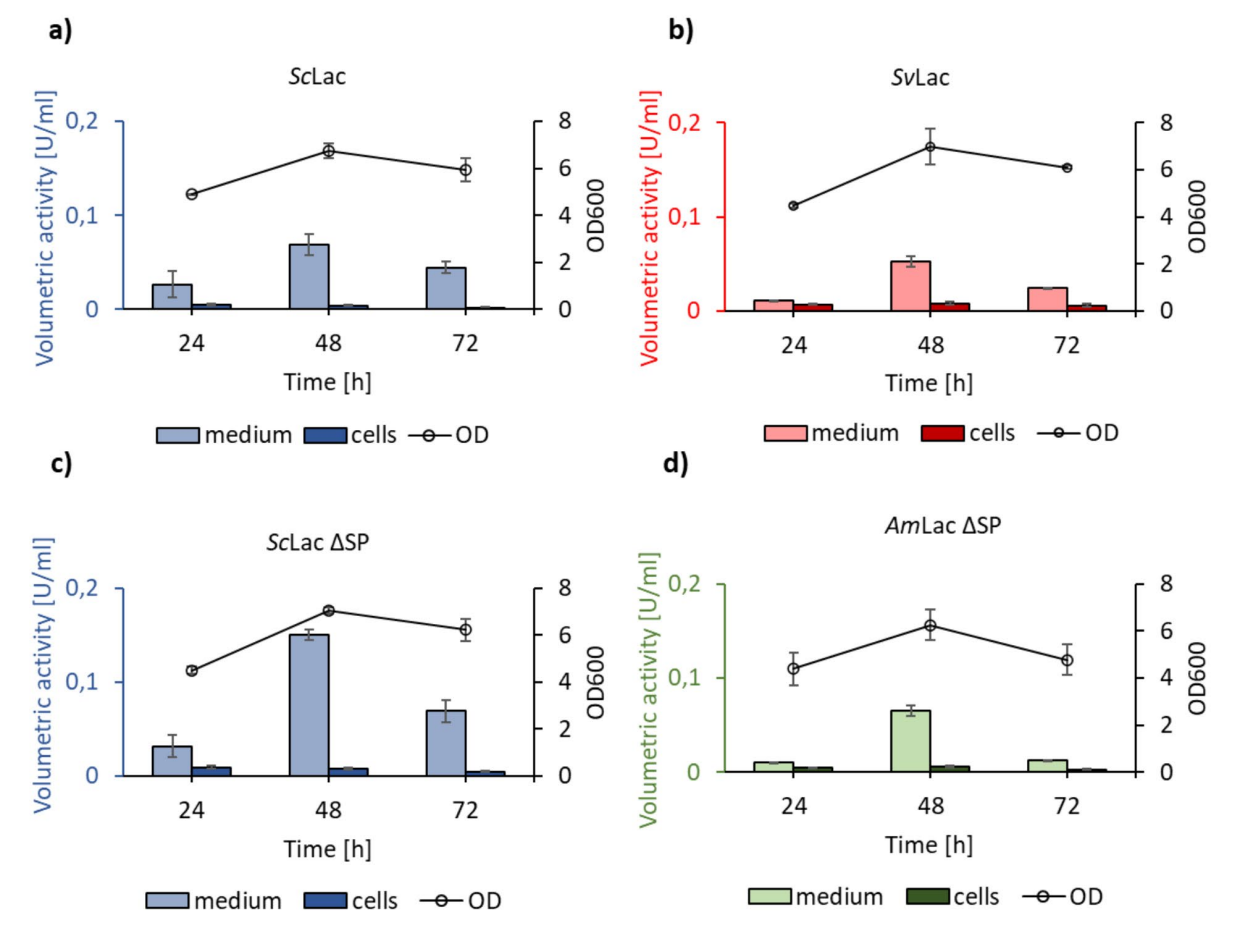
Expression of the small laccases genes in *B. subtilis* RIK1285 host organism. Secretion of **a) ***Sc*Lac, **b) ***Sv*Lac, **c) ***Sc*Lac ∆SP and **d) ***Am*Lac ∆SP. Bacteria were inoculated in the growth media containing 100 µM of CuSO_4_. The samples were taken at defined time points, cells were separated by centrifugation and activity was measured using ABTS as a substrate. The bacterial growth was measured spectrophotometrically at 600 nm. *Sc*Lac and *Sv*Lac contained signal sequences at the N terminus, whereas in *Am*Lac ∆SP and *Sc*Lac ∆SP the signal sequence was deleted. *Sc*Lac and *Sv*Lac were secreted with their signal sequence still attached. *Sc*Lac ∆SP and *Am*Lac ∆SP were secreted without signal sequences. The activity results were the average of three biological replicates in three technical replicates each

Since *B. subtilis* NZ8900 did not have any of the endogenous proteases deleted, degradation of secreted laccases may be responsible. To address this problem, we expressed all three small laccases genes under control of a constitutive promoter in the *B. subtilis* strain RIK1285 *nprR2*, *nprE18* (Table 1). The time course of laccase activity was measured in the presence of copper. Extracellular activity was measured for *Sc*Lac and *Sv*Lac after 24 h (Fig. 5a and b) and reached a peak after 48 h. Only low residual activity was measured in cell extracts, indicating successful secretion. No extracellular or intracellular activity could be measured for *Am*Lac.

The mass-spectrometry analysis of the secreted *Sc*Lac and *Sv*Lac in *B. subtilis* detected peptides corresponding to the signal sequence (Supplementary Figs. 4 and 5). This indicated that *Sc*Lac and *Sv*Lac were secreted in *B. subtilis* RIK1285 but were not properly processed. Unexpectedly, significant extracellular activity for *Am*Lac ∆SP and *Sc*Lac ∆SP samples with deleted signal sequences was measured after 24 h (Fig. 5c and d), again with a peak after 48 h. No extracellular or intracellular activity could be measured for the *Sv*Lac ∆SP sample. The significant difference in extracellular and intracellular activity suggested the secretion of the small laccases without signal sequence.

To study the effect of copper on small laccase secretion in *B. subtilis*, the enzymes were expressed in the absence of copper supplementation (Fig. 2a). Only the samples with initial copper supplementation showed extracellular activity (Fig. 5). The reconstitution of the secreted enzyme activity was unsuccessful.

### The secretion of the recombinant small laccases in *B. subtilis* was not due to cell lysis

**Fig. 6:**
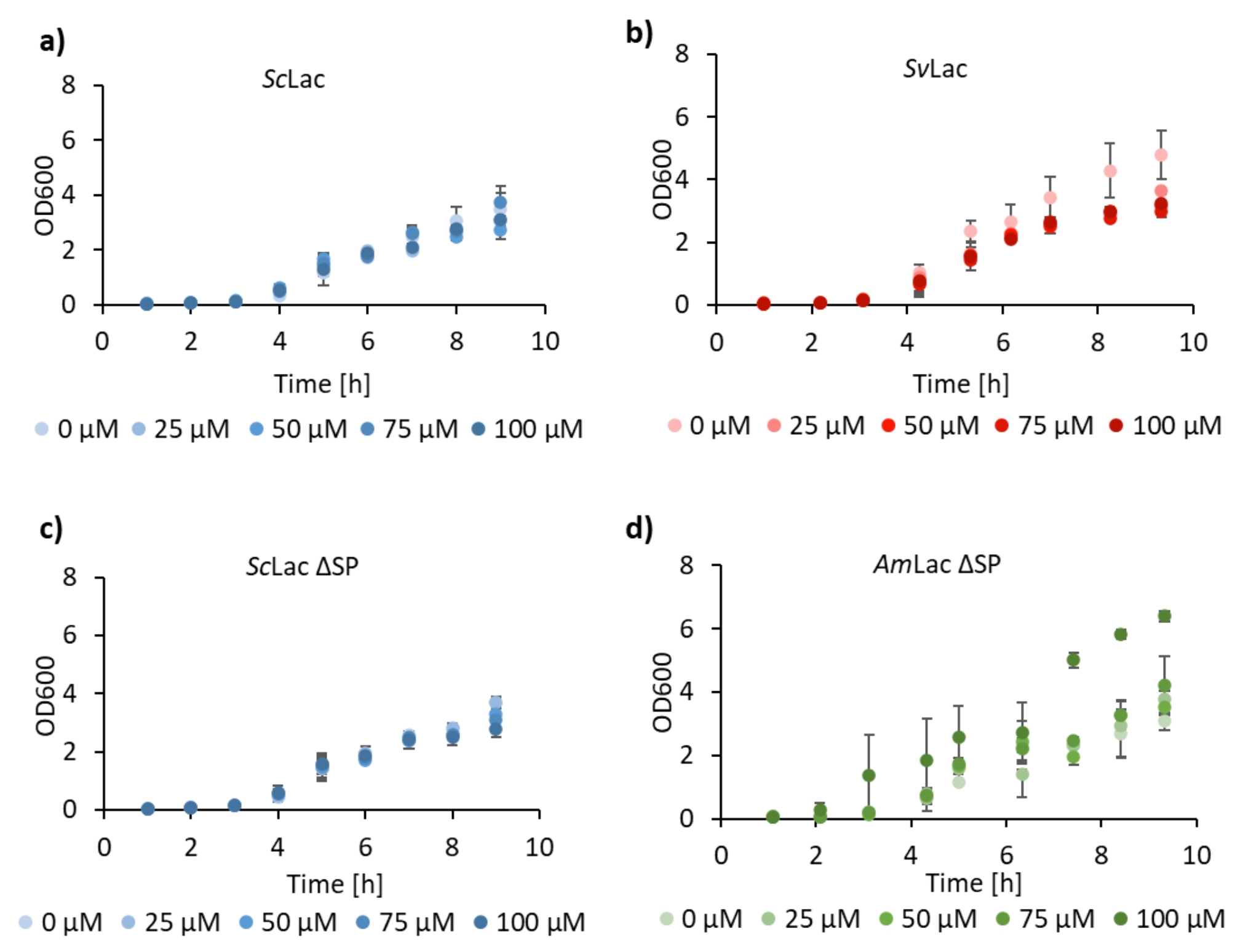
Early growth of *B. subtilis* RIK1285 producing small laccases with different concentrations of copper. The optical density of *B. subtilis* RIK1285 expressing **a) ***Sc*Lac, **b) ***Sv*Lac, **c) ***Sc*Lac ∆SP and **d) ***Am*Lac was measured at 600 nm every hour for nine hours with CuSO_4_ concentrations ranging from 0 to 100 µM. The copper had no detrimental effect on the growth of *B. subtilis* that expressed and secreted small laccases. The results were the average of three biological replicates

The addition of excess copper in the growth medium could lead to cell lysis and explain the extracellular activity. Therefore, we supplemented the growth medium with different copper concentrations and followed *Am*Lac, *Sc*Lac, *Sc*Lac ∆SP and *Sv*Lac growth in the early exponential phase. As shown in Fig. 6 there was no significant difference in growth among the strains and copper concentrations, supporting that copper had no detrimental effect.

To support the argument against cell lysis, viability was assessed with propidium iodine (PI). PI is a reagent that binds to DNA but is excluded when the membrane is intact [51]. *B. subtilis* cells producing *Sc*Lac, *Sc*Lac ∆SP, and *Am*Lac ∆SP were collected after 48 h when the extracellular activity reached a maximum, stained with PI and visualized by fluorescence microscopy. There was no significant difference in cell integrity when growth medium was supplemented with copper (Supplementary Fig. 6) indicating that copper had no detrimental effect on viability.

Finally, another type of enzyme, *Amycolatopsis* 75iv2 dye-decolorizing peroxidase (*Am*DyP) was used to study the effect of copper supplementation on cell viability. The gene encoding *Am*DyP was expressed constitutively in the *B. subtilis* strain RIK1285 *nprR2*, *nprE18* and cultivated with 100 µM CuSO_4_ in the growth medium. No *Am*DyP activity was measured in the culture supernatant, but activity could be detected in the cell extracts (Supplementary Fig. 7).

## Discussion

In this study we used *S. lividans* and *B. subtilis* as host organisms for extracellular production of three small laccases using their native signal peptides. The small laccases were predicted to be secreted via the Tat-pathway, and their signal peptides all contained the characteristic twin arginine motif (Fig. 1). In *S. lividans* around 21% of the secretome is predicted to be translocated via the Tat-pathway [52]. The Tat-pathway generally handles proteins with cofactors that are folded into their functional conformation in the cytoplasm prior to translocation [45] including small laccases, heme-containing peroxidases [53] or flavoproteins [39]. All three genes encoding small laccases, *Sc*Lac, *Sv*Lac and *Am*Lac, were successfully expressed and secreted, albeit with different titers of 1950 ± 99 U/l, 812 ± 57 U/l and 12 ± 1 U/l, respectively. Majumdar and colleagues characterized small laccases expressed in *E. coli* and purified from the cell extracts using lignin model compounds as substrates and reported three times lower k_rel_/K_app_ for *Am*Lac compared to *Sc*Lac [20], indicating that the enzyme may generally have a lower activity. Additionally, *Amycolatopsis* spp. are phylogenetically more distant to the expression host *S. lividans* than *S. coelicolor* and *S. viridosporus*, which may lead to suboptimal folding and secretion of *Am*Lac. Mass-spectrometric analysis of the secreted proteins confirmed correct processing (removal of the signal peptide post-translocation) for *Sc*Lac. *Sc*Lac was expressed and secreted in *S. lividans* previously [54, 55]. In their studies *Sc*Lac gene was amplified from the GC-rich genome and expressed in *S. lividans*, whereas in our study codon optimized *Sc*Lac was successfully expressed in *S. lividans*. *Sc*Lac was also produced in *P. pastoris* using the *S. cerevisiae* α factor signal sequence [56]. The activity in the *P. pastoris* supernatant reached 500 ± 10 U/l after 5 days, which is approximately 25% of the volumetric yield achieved here after 3 days. Secretory expression of *Sc*Lac was also reported in *A. oryzae*, but without details on activity or yield [57]. Secretory production of *Am*Lac and *Sv*Lac was not previously reported to our knowledge.

We also expressed the three laccase-encoding genes without their signal peptides. *Sc*Lac ∆SP only showed activity in the medium after 96 h of cultivation, indicating that cell lysis is responsible for the release of the intracellularly accumulated enzyme. For unknown reasons, *Sv*Lac ∆SP did not show any activity in either cell extract or supernatant. *Sv*Lac ∆SP was previously produced cytoplasmatically in *E. coli* using the gene amplified from the genome [20], but our construct *Sv*Lac ∆SP did also not result in intracellular accumulation of active enzyme. Transformation of *Am*Lac ∆SP construct in *S. lividans* was unsuccessful for unknown reasons.

Laccases are multicopper enzymes and therefore we investigated the role of copper supplementation in secretion. Our results indicate that *Am*Lac, *Sc*Lac and *Sv*Lac were secreted in the *S. lividans* expression system irrespective of additional copper supplementation. However, significantly higher activity of the secreted enzymes was observed when copper ions were present at the beginning of the expression (Fig. 4d, e and f), and reconstitution of active enzyme did not reach the same activity levels. These results suggest that apoenzymes could be prone to degradation due to improper folding, or that the incorporation of the copper ions into folded and secreted enzymes does not happen quantitatively. We speculate that the incorporation of copper into the T2 position is particularly difficult because of its position at the end of a long and narrow channel [58]. This should be considered for small laccases production. In addition, microbial growth conditions play important role in fully loaded laccases. Microaerobically grown *E. coli* accumulated higher amounts of copper than aerobically grown *E. coli*, thus resulting in fully active enzyme [59].

We also expressed the *Am*Lac, *Sc*Lac and *Sv*Lac genes in *B. subtilis*. No activity in the culture supernatants could be observed in the initial experiments using the SURE system and the strain NZ8900. This is in agreement with recent observations with a flavin-dependent pyranose oxidase from *K. aureofaciens* [39] and is most likely due to high extracellular protease activity degrading the secreted heterologous enzymes. Using the strain RIK1285 and a constitutive expression system, extracellular activity was measured for *Sc*Lac and *Sv*Lac (Fig. 5a and b). No extracellular or intracellular activity was observed for *Am*Lac. It appears plausible that the lower activity reported by Majumdar and colleagues as well as suboptimal folding and secretion as observed in the expression in *S. lividans* (this work) result in activity below the detection limit [20]. The mass-spectrometry analysis of secreted *Sc*Lac and *Sv*Lac revealed the presence of peptides representing the signal sequences, indicating that the enzymes were not properly processed during translocation. *S. lividans* contains four signal peptidases (SPases) [60], and a strain deficient in one of them (sipY) was shown to secrete only very few proteins [61]. *B. subtilis* contains five SPases but no homologue of SipY [62], which could be the reason that the signal sequences of *Streptomyces* spp. are not cleaved after translocation.

To our great surprise, cultivation of *B. subtilis* producing the constructs *Am*Lac ∆SP and *Sc*Lac ∆SP also resulted in extracellular activity. Several previous studies showed recombinant protein secretion in *B. subtilis* via (a) non-classical secretion pathway(s) [63–68]. Each of these studies used different *B. subtilis* expression host (*B. subtilis* 168, 1A751, WB800 and WB600BHM). In this study, *B. subtilis* NZ8900 and *B. subtilis* RIK1285 were used, and only in the latter secreted activity could be observed. Two studies about the non-classical secretion showed that proteins could be secreted due to their multimerization and hydrophobicity [64, 66]. *Am*Lac (PDB ID: 3TA4), *Sc*Lac (PDB ID: 3CG8) and *Sv*Lac (PDB ID: 3TBC) are trimeric enzymes, thus intracellular folding into a trimeric structure may be involved. Secretome studies of *B. subtilis* revealed 24 cytoplasmic proteins without the typical signal sequences in the medium [69]. Thus, *B. subtilis* secretes some native and recombinant proteins lacking signal sequence via an unknown pathway.

The viability assays did not show copper-dependent growth inhibition or cell lysis, indicating *B. subtilis* robustness against excess copper ions in the tested concentrations. Addition of copper after production in *B. subtilis* did not reconstitute enzyme activity as it did with laccases secreted by *S. lividans*. We assume that the low volumetric activities (which were significantly lower than in *S. lividans)* for all tested enzymes in *B. subtilis* and thus the presumably low rate of inactive apo-enzymes is responsible for this.

## Methods and materials

### Bacterial strains, plasmids and growth conditions

The bacterial strains and plasmids used in this study are listed in Table 1. *Am*Lac (WP_020416648.1), *Sc*Lac (WP_003972284.1) and *Sv*Lac (WP_016823706.1) genes were codon optimized for *B. subtilis* host organism and synthesized by Twist Bioscience (USA). *Amycolatopsis* 75iv2 dye-decolorizing peroxidase (*Am*DyP) gene (WP_020421762.1) was codon optimized for *E. coli* and synthesized by General Biosystems (USA). *B. subtilis* NZ8900, RIK1285, and *S. lividans* TK24 were used as host organisms for protein expression. *E. coli* MC1061, JM109 were used as host organisms for intermediate cloning. Plasmid pNZ8901 was used in the *B. subtilis* NZ8900 expression system. Plasmid pBE-S was used in the *B. subtilis* RIK1285 expression system. Plasmid pIJ486 was used in the *S. lividans* expression system. *E. coli* and *B. subtilis* transformants were grown in 2xYT medium (16 g/l tryptone; 10 g/l yeast extract; 5 g/l NaCl) supplemented with ampicillin (100 µg/ml), chloramphenicol (10 µg/ml or 5 µg/ml) or kanamycin (10 µg/ml) depending on the antibiotic marker of the plasmid. *S. lividans* transformants were grown in phage medium (0.5 g/l MgSO_4_ 7H_2_O; 0.74 g/l CaCl_2_ 2H_2_O; 10 g/l glucose; 5 g/l tryptone; 5 g/l yeast extract, 5 g/l Lab Lemco powder, pH 7.2) supplemented with 10 µg/ml thiostrepton. *B. subtilis* NZ8900 strain and *S. lividans* strains were incubated at 30 °C 160 rpm. *B. subtilis* RIK1285 and *E. coli* strains were incubated at 37 °C 160 rpm. All experiments were conducted in triplicates.

### Primers, cloning and transformation

Polymerase chain reaction (PCR) primers used in this study are listed in the Supplementary Table 1. Primers were synthesized by Microsynth AG (Switzerland). PCRs were performed using Q5 High Fidelity DNA Polymerase (New England Biolabs, USA) according to the manufacturer’s protocol. The PCR products were purified using Monarch PCR & DNA Cleanup Kit (New England Biolabs, USA). Plasmids containing pNZ8901 or pBE-S backbone were cloned using Gibson assembly (NEBuilder HiFi DNA Assembly Master Mix, New England Biolabs, USA). The plasmid pNZ8901 was linearized with *Nco*I and the plasmid pBE-S was linearized by PCR using primer pair 17 (Supplementary Table 1). The inserts were amplified with the primers containing complementary overhangs to the backbone (overhang sequences highlighted in Supplementary Table 1). The linearized backbones and inserts with overhangs were incubated for 30 min at 50 °C and immediately transformed into chemically competent *E. coli. B. subtilis* NZ8900 and *B. subtilis* RIK1285 were transformed by electroporation or natural competence as described in manufacturers’ protocols (MoBiTec, Germany; Takara Bio, Japan). Plasmids were extracted using Monarch Plasmid Miniprep Kit (New England Biolabs, USA).

The pUC19-P_vsi_ plasmid was used as an intermediate backbone for cloning pIJ486 plasmids. The pUC-P_vsi_ plasmids were cloned using Gibson assembly. pUC19-P_vsi_ was linearized with *Pst*I, incubated with the inserts containing overhangs to the backbone for 30 min at 50 °C and transformed into *E. coli*. The pUC19-P_vsi_ plasmids containing inserts and pIJ486 backbone were both digested with *Hin*dIII and *Xba*I, excised from 0.8% agarose gel using Monarch DNA Gel Extraction Kit (New England Biolabs, USA) and ligated using T4 DNA ligase according to manufacturer’s protocol (New England Biolabs, USA). The ligation mixture was directly transformed into *S. lividans* protoplasts as described in [70]. The correct sequences of all DNA constructs were confirmed by Sanger sequencing (Microsynth AG, Switzerland).

### Protein expression

Protein production in *B. subtilis* NZ8900 was induced according to the manufacturer’s protocol. Briefly, *B. subtilis* NZ8963 was grown overnight and the next day were inoculated into a fresh medium at optical density at 600 nm (OD) 0.15. The supernatant at OD 1.0 was collected and heated for 10 min at 80 °C. The supernatant of *B. subtilis* NZ8963 served as an inducing agent containing subtilin. *B. subtilis* NZ8900 strains were cultivated overnight and 1/100 was inoculated into a fresh medium. When the culture reached at OD 0.7–0.8 then gene expression was induced with 2% v/v subtilin. *B. subtilis* RIK1285 strains were grown overnight and the next day 1/100 of the preculture was inoculated into a fresh medium. Proteins were produced under the constitutive promoter aprE. *S. lividans* spores were prepared as described in [70]. *Am*Lac (1000 CFU/ml), *Sc*Lac (10 000 CFU/ml) and *Sv*Lac (10 000 CFU/ml) spores were inoculated directly into 50 ml of phage medium cultivated in the 300 ml baffled flasks. Proteins were produced under the constitutive promoter P_vsi_. One ml of the culture was harvested at 24, 48, 72 h for *B. subtilis* strains and at 24, 48, 72, 96 (and 120 h in the case of *Am*Lac) hours for *S. lividans* strains. The samples were centrifuged for 15 min at 4 °C 10 000 rpm. The pellet was washed twice with 0.9% NaCl solution, centrifuged for seven minutes at 4 °C 15 000 rpm and resuspended in the initial sample volume one ml of 20 mM Tris-HCl pH 7.4 buffer. The cells were lysed using Sonopuls HD60 (Germany) 40–50 s 50 cycles 80% power and centrifuged for 7 min at 4 °C 15 000 rpm. The supernatant and cell extract were used in the activity assays.

*B. subtilis* RIK1285 expression medium was supplemented with 0, 25, 50, 75 or 100 µM CuSO_4,_ harvested at every hour for nine hours and measured OD at 600 nm to study the effect of the CuSO_4_ on the growth. *S. lividans* phage medium was supplemented with 0 and 100 µM CuSO_4_, harvested at selected time points and wet cell weight (WCW) was measured as the growth parameter.

### Copper reconstitution assay

Protein production was conducted in 50 ml of the medium containing 0 or 100 µM CuSO_4_. At the selected time points the samples were harvested as described above. The supernatant and cell extract that contained 0 µM CuSO_4_ at the start of the production were supplemented with 100 µM CuSO_4,_ incubated at 4 °C for 16 h and measured activity as described below. The samples where CuSO_4_ was added later were named medium (R) and cells (R).

### Laccase activity assay

Small laccase activity was measured using 2,2′-azinobis(3-ethylbenzothiazoline-6-sulfonate) (ABTS) as the substrate. The oxidation of ABTS was detected by measuring the absorbance at 420 nm (ε420 = 36,000 M − 1 cm − 1). The reaction mixture (300 µl) contained 60 µl of 50 mM ABTS (final concentration 10 mM), 60 µl of enzyme (supernatant or cell extract) and 180 µl of 100 mM sodium citrate pH 4 buffer. ABTS was prepared fresh prior to each measurement. The reactions were measured using plate reader EnSpire multimode (Perkin Elmer, USA). The supernatant and cell extract from strains containing empty plasmid and growth medium were used as negative controls.

### Dye-decolorizing peroxidase activity assay

Dye-decolorizing peroxidase (DyP) activity was measured using ABTS as the substrate. The oxidation of ABTS was detected by measuring the absorbance at 420 nm (ε_420_ = 36,000 M^− 1^ cm^− 1^). The reaction mixture (300 µl) contained 30 µl of 25 mM ABTS (final concentration 2.5 mM), 60 µl of enzyme (supernatant or cell extract), 30 µl of 5 mM H_2_O_2_ (final concentration 0.5 mM) and 180 µl of 50 mM sodium acetate pH 4.5 buffer. ABTS and H_2_O_2_ solutions were prepared fresh prior to each measurement. The reactions were measured using plate reader EnSpire multimode (Perkin Elmer, USA). The supernatant and cell extract from strains containing empty plasmid and growth medium were used as negative controls.

### Mass-spectrometry analysis

The supernatants of *Sc*Lac and *Sv*Lac were collected at 48 h and were ran on SDS PAGE (Bio-Rad, USA) at 120 V for one hour using Precision Plus Protein™ Unstained Protein Standard (Bio-Rad, USA). The gel was incubated with Coomassie stain (Bio-Rad, USA) at 30 °C for one hour and washed several times with 10% ethanol and 2% acetic acid solution. The bands of *Sc*Lac and *Sv*Lac were excised at the expected sizes 35 kDa and 36 kDa, respectively and subjected to digestion. The samples were digested in-solution. The proteins were S-alkylated with iodoacetamide and digested with Trypsin (Promega). The digested samples were loaded on a C18 column (ACQUITY PRM HSST3 1.8µ 2.1 × 150 mm, Waters) using 0.1% formic acid as the aqueous solvent. A gradient from 3.5% B (B: 80% ACN, 20% A) to 40% B in 30 min was applied, followed by a 5 min gradient from 40% B to 95% B that facilitates elution of large peptides, at a flow rate of 250 µL/min. Detection was performed with a Q-TOF instrument (Agilent Series 6560 LC-IMS-QTOFMS) equipped with the Jetstream ESI source in positive ion, DDA mode (switching to MSMS mode for eluting peaks). MS-scans were recorded (range: 400–3200 Da) and the 5 highest peaks were selected for fragmentation. Instrument calibration was performed using ESI calibration mixture (Agilent).

### Fluorescence microscopy

One ml of the *B. subtilis* RIK1285 cultures producing *Sc*Lac and *Am*Lac ∆SP were collected at 48 h, washed twice with Phosphate-buffered Saline pH 7.4 (PBS) and diluted with PBS at OD 0.4 with final volume of 199 µl. The cells were stained with one µl Propidium Iodine (PI 1 mg/ml, Biotium) and subjected to fluorescence microscopy. The fluorescence microscopy and image acquisition were carried out using live-cell epi-fluorescence Leica DMI6000B (Germany) equipped with HCX PL APO 63x/1.40 oil objective and Leica N2.1 filter cube (BP 515–560). The images were acquired using DFC360FX camera resulting in 1392 × 1040 pixels. The unstained cells were used as negative control. The percentage of dead cells was calculated by counting PI-stained cells and unstained cells, and dividing PI-stained cells with total number of the cells.

## Conclusion

The expression of small laccases genes were studied in *S. lividans* and *B. subtilis*. *S. lividans* poses more difficulties in handling (e.g., transformation procedures) and cultivation, generally grows slower than *B. subtilis*, and there is a smaller choice of established promoters, plasmid selection markers or signal peptides. However, the volumetric activities reached in these cultures were significantly higher for the enzymes produced in *S. lividans*. If alternative *B. subtilis* strains with fewer extracellular protease activity are used and expression can be further optimized, the picture may be different, but the correct processing of the secretory proteins (removal of signal peptide post-translocation) in *S. lividans* has to be considered advantageous.

## Electronic supplementary material

Below is the link to the electronic supplementary material.

**Additional file 1: Supplementary figure 1.** SignalP 5.0 predictions of the studied small laccases. **Supplementary figure 2.** The earlygrowth of *S. lividans* and secretion studies of *Sc*Lac ΔSP. **Supplementary figure 3.** The detection of *Sc*Lac signal sequence peptides in *S. lividans* expression system. **Supplementary figure 4.** The detection of *Sc*Lac signal sequence peptides in *B. subtilis* RIK1285 expression system. **Supplementary figure 5.** The detection of *Sv*Lac signal sequence peptides in *B. subtilis *RIK1285 expression system. **Supplementary figure 6.** The viability of *B. subtilis * RIK1285 expressing recombinant small laccese genes using fluorescent microscopy. **Supplementary figure 7.***Am*DyP production in *B. subtilis* RIK1285. **Supplementary figure 8.** An overview of the plasmids used in this study. **Table 1.** Primers used in this study.

## Data Availability

All data generated or analyzed during this study are included in this article and the supplementary files. Materials are available from the corresponding author on reasonable request.
