# Supplementary material for: Secretory expression of recombinant small laccase genes in Gram-positive bacteria
Source: Microb Cell Fact. 2023 Apr 17;22:72. doi: 10.1186/s12934-023-02075-5 (PMC10108450; doi:10.1186/s12934-023-02075-5)
Supplement: Supplementary file 1 — Additional file 1: Supplementary figure 1. SignalP 5.0 predictions of the studied small laccases. Supplementary figure 2. The earlygrowth of S. lividans and secretion studies of ScLac ΔSP. Supplementary figure 3. The detection of ScLac signal sequence peptides in S. lividans expression system. Supplementary figure 4. The detection of ScLac signal sequence peptides in B. subtilis RIK1285 expression system. Supplementary figure 5. The detection of SvLac signal sequence peptides in B. subtilis RIK1285 expression system. Supplementary figure 6. The viability of B. subtilis RIK1285 expressing recombinant small laccese genes using fluorescent microscopy. Supplementary figure 7.AmDyP production in B. subtilis RIK1285. Supplementary figure 8. An overview of the plasmids used in this study. Table 1. Primers used in this study. [file 12934_2023_2075_MOESM1_ESM.docx]

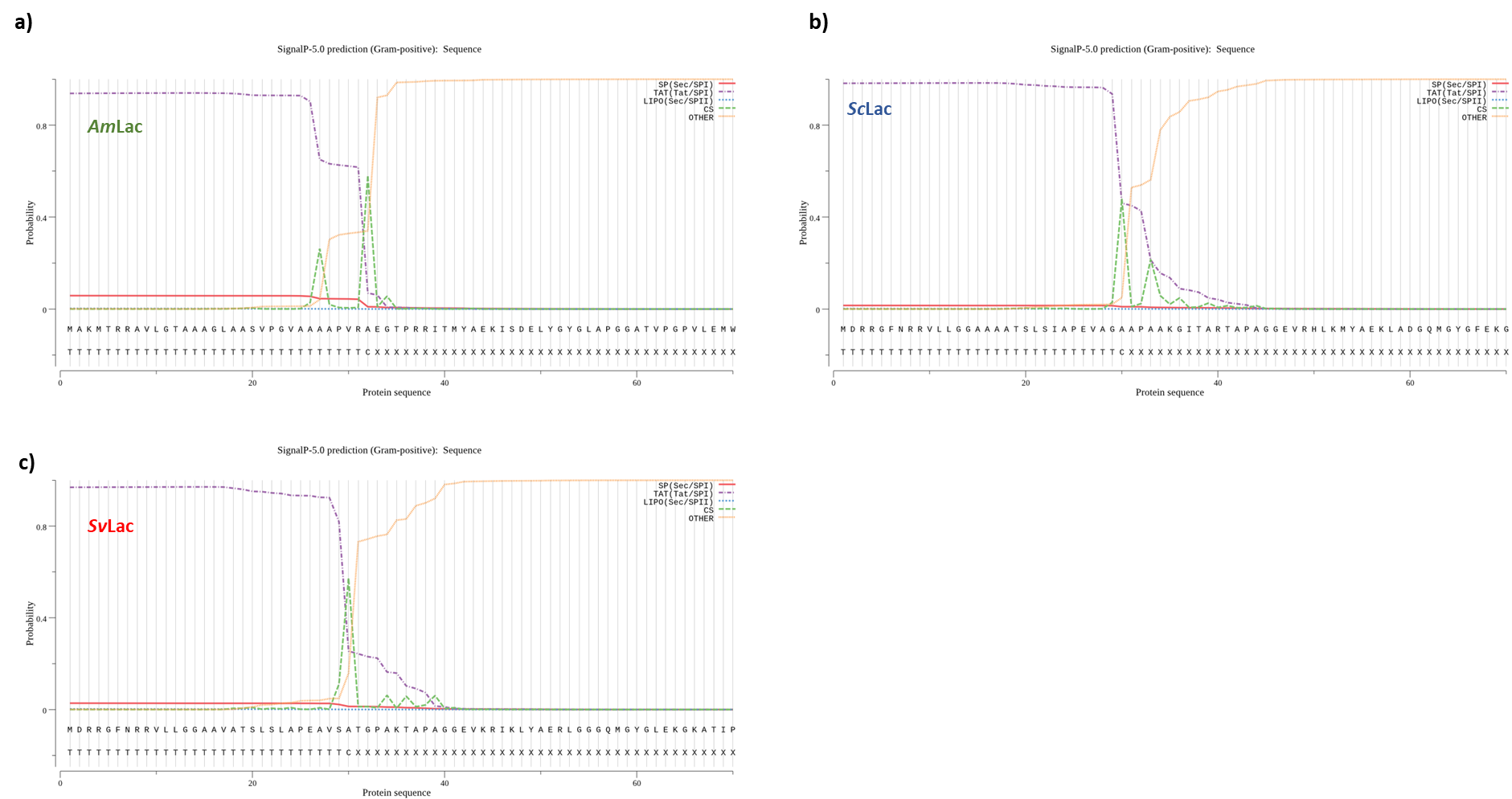


**Supplementary figure 1: SignalP 5.0 predictions of the studied small laccases**. **a)** AmLac, **b)** ScLac, and **c)** SvLac have predicted tat-pathway signal sequence (shown in dashed purple line). The peptidase cutting site for AmLac was predicted between VRA and EG, for ScLac between AGA and AP and for SvLac between VSA and TG amino acids. The predicted cutting sites were considered when the constructs without signal sequence (ScLac ∆SP, AmLac ∆SP, SvLac ∆SP) were constructed.


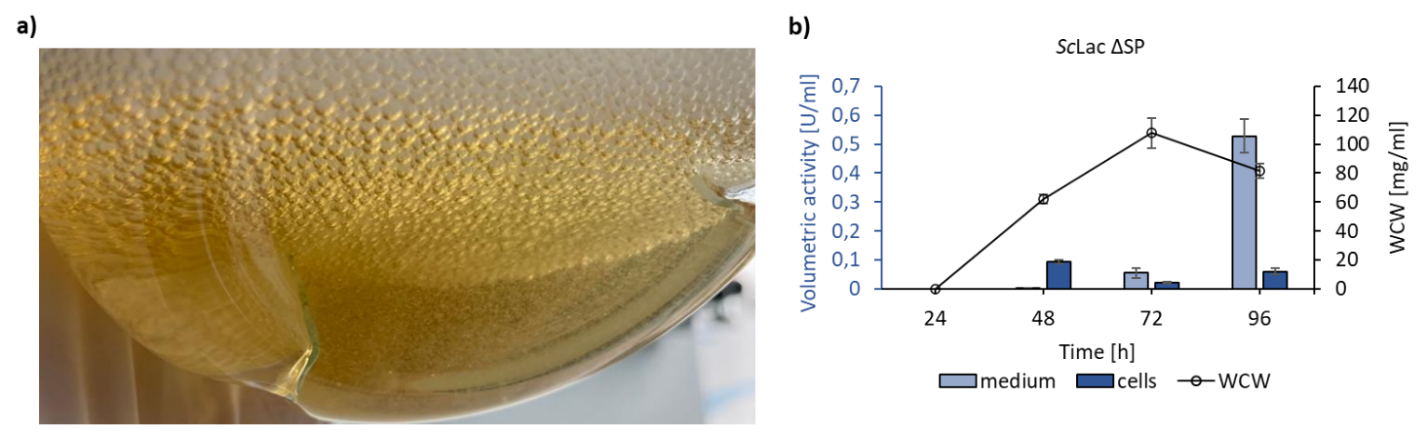


**Supplementary figure 2: The early growth of S. lividans and secretion studies of ScLac ∆SP.** **a)** The spores of ScLac after 24 hours. The spores of S. lividans expressing AmLac, ScLac and SvLac had similar morphology that resembled to white flakes. No samples were taken due to the minimal cell growth.


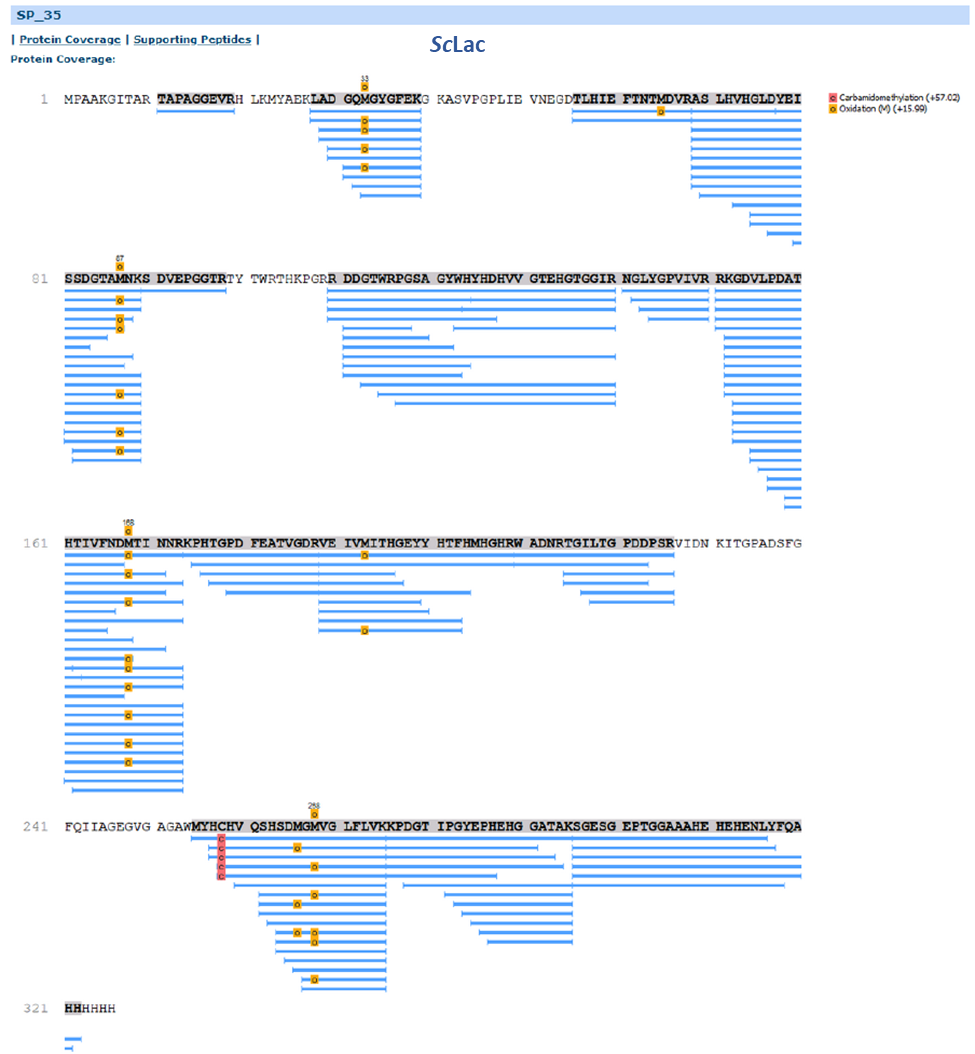


**Supplementary figure 3: The detection of ScLac signal sequence peptides in S. lividans expression system**. Amino acids sequences identified by MS/MS were highlighted in grey. Blue lines indicated individual peptides identified. No peptide from the signal sequence MDRRGFNRRVLLGGAAAATSLSIAPEVAGAAP was detected indicating that enzyme was processed.


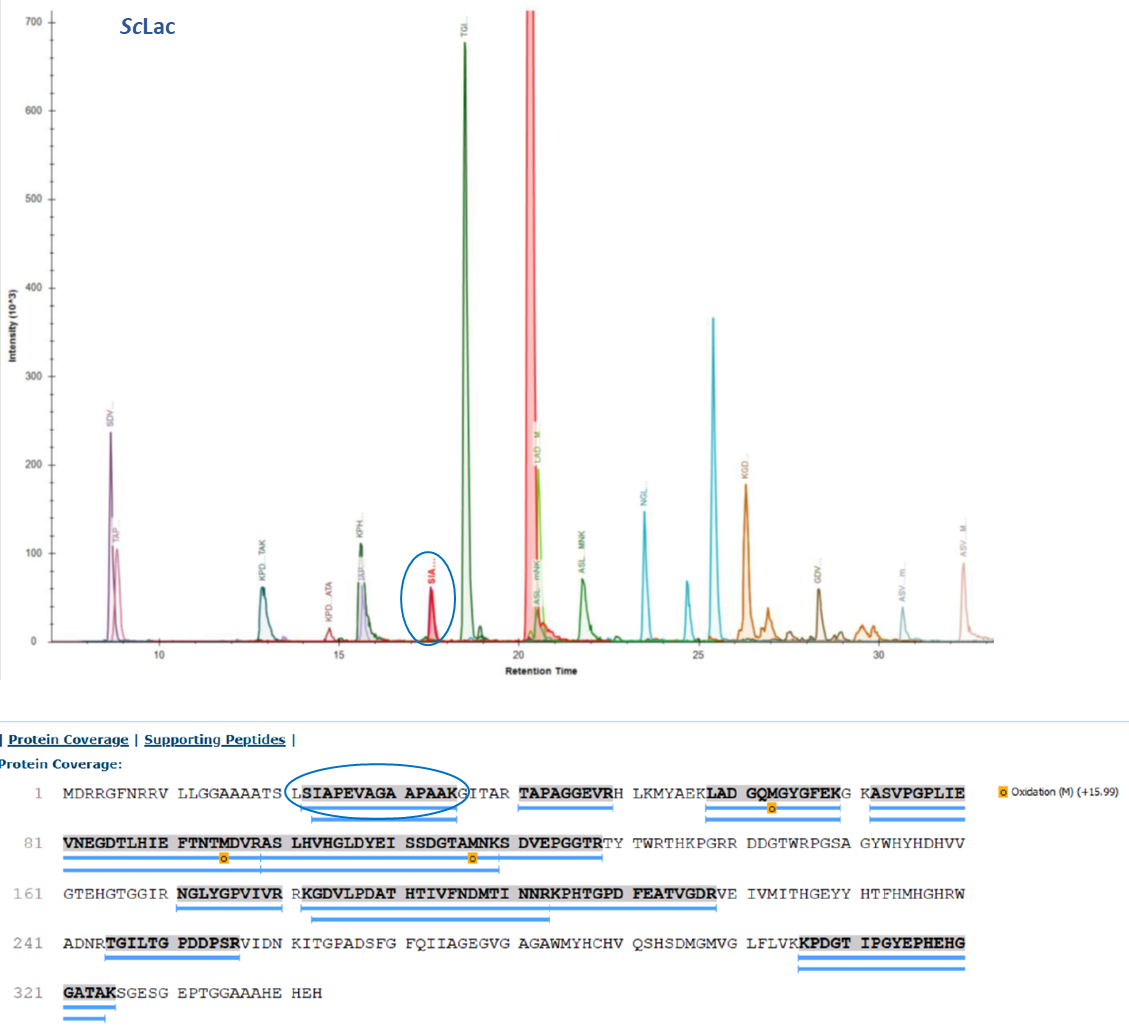


**Supplementary figure 4: The detection of ScLac signal sequence peptides in B. subtilis RIK1285 expression system.** Amino acids sequences identified by MS/MS were highlighted in grey. Blue lines indicated individual peptides identified. A peptide from the signal sequence MDRRGFNRRVLLGGAAAATSLSIAPEVAGAAP was detected indicating that enzyme was not properly processed. The detected peptide from the signal sequence was circled in blue.


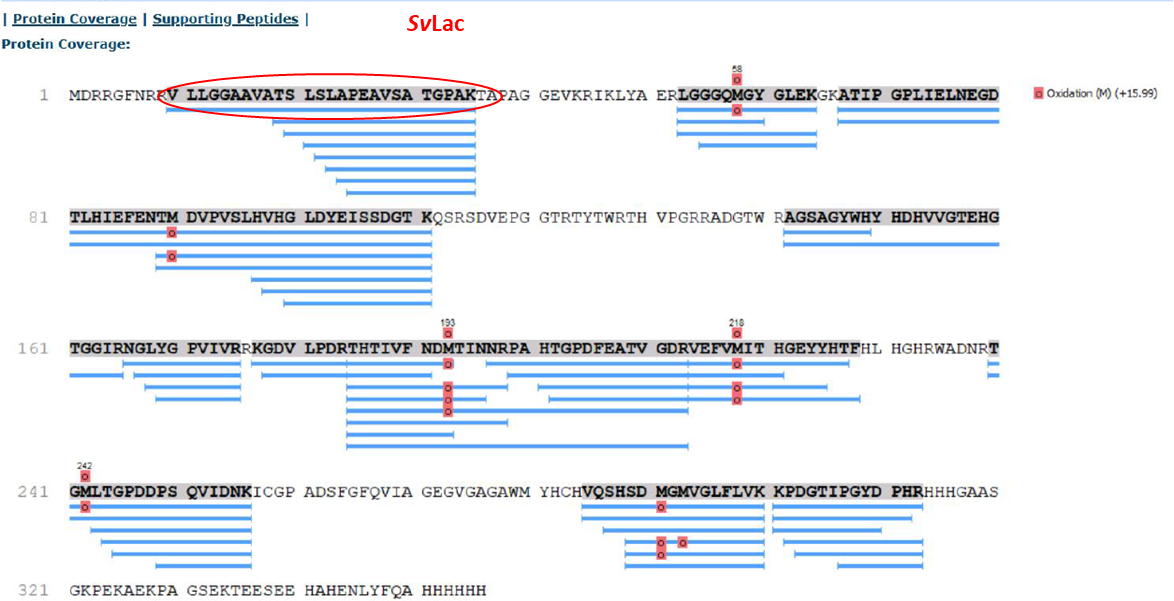


**Supplementary figure 5:** **The detection of SvLac signal sequence peptides in B. subtilis RIK1285 expression system.** Amino acids sequences identified by MS/MS were highlighted in grey. Blue lines indicated individual identified peptides. A peptide from the signal sequence MDRRGFNRRVLLGGAAVATSLSLAPEAVSATG was detected indicating that enzyme was not properly processed. The detected peptide from the signal sequence was circled in red.


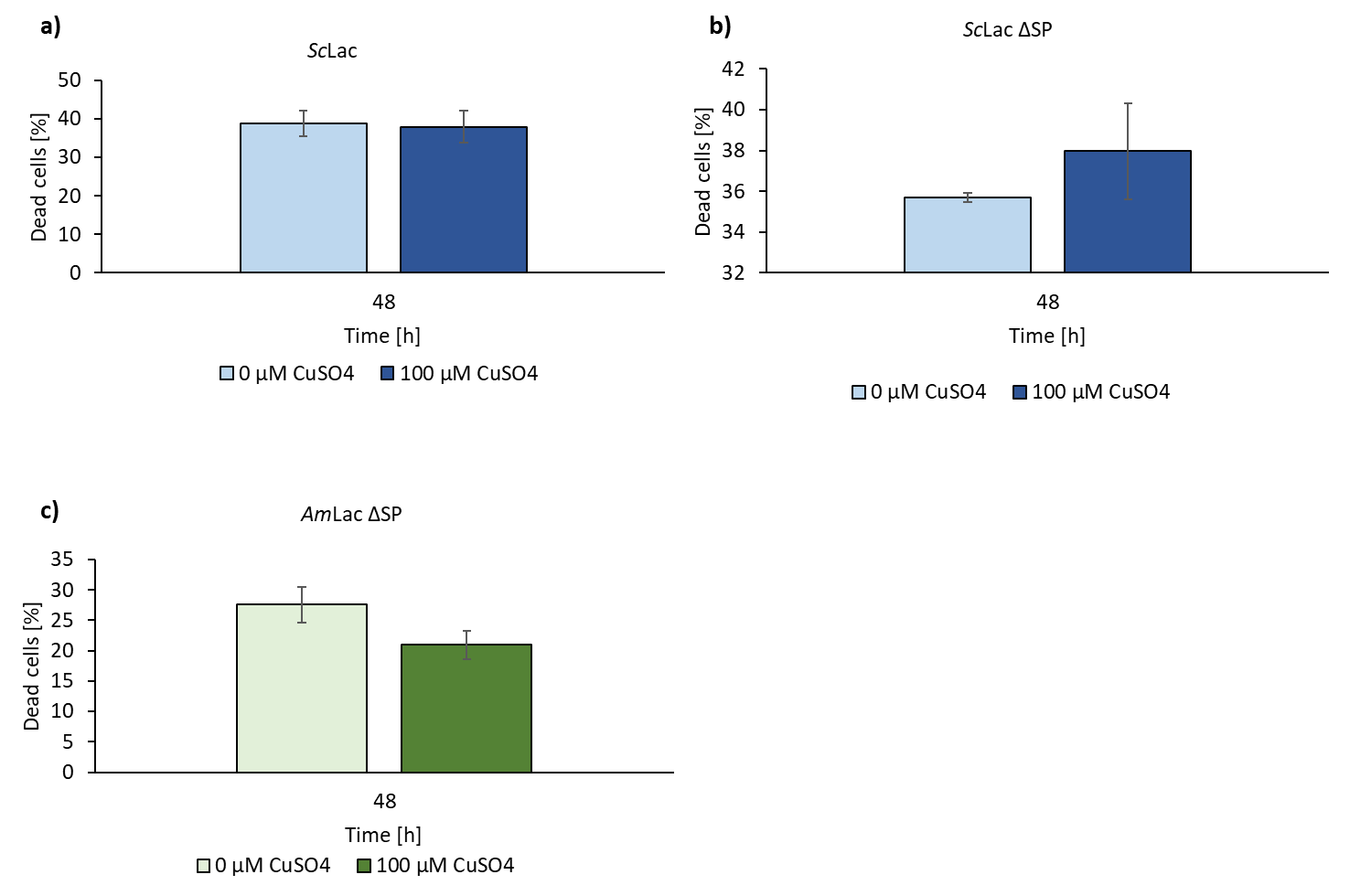


**Supplementary figure 6: The viability of B. subtilis RIK1285 expressing recombinant small laccases genes using fluorescence microscopy**. The percentage of the dead cells of **a)** ScLac, **b)** ScLac ∆SP, and **c)** AmLac ∆SP after 48 hours in the presence or absence of copper. The cells were stained with PI that only penetrated the cells with compromised membrane integrity. There was no significant difference of dead cells when copper was present indicating harmless effect on the growth. Three pictures of different areas for each construct were taken and minimum 75 cells per picture were counted.


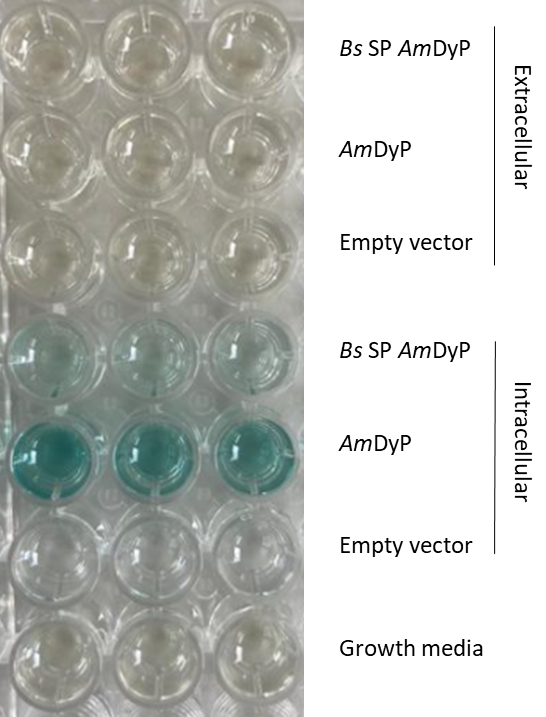


**Supplementary figure 7: AmDyP production in B. subtilis RIK1285.** Dye-decolorizing peroxidase from Amycolatopsis 75iv2 (AmDyP) was cloned in the pBE-S plasmid and transformed into B. subtilis RIK1285 expression host. The expression medium was supplemented with copper. The activity was measured using ABTS as a substrate but only detected inside of the cells. This indicated that excess copper in the growth medium did not induce cell lysis. Empty plasmid and growth medium were used as the negative controls.


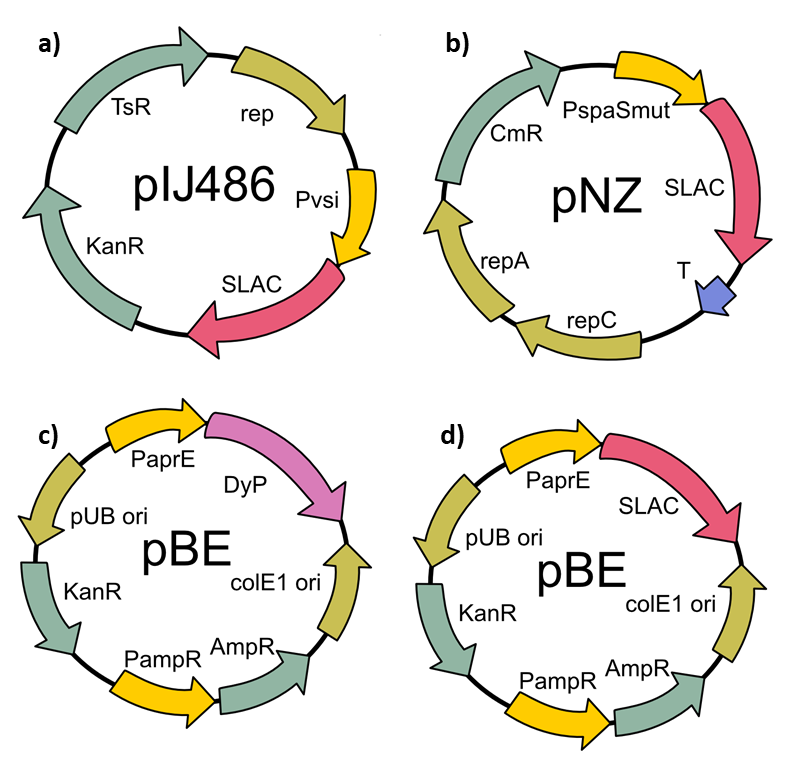


**Supplementary figure 8: An overview of the plasmids used in this study.** **a)** pIJ486 plasmid for S. lividans expression system containing AmLac, ScLac, ScLac ∆SP, SvLac, and SvLac ∆SP genes. **b)** pNZ8901 plasmid for B. subtilis NZ8900 expression system containing ScLac and ScLac ∆SP genes. **c)** pBE-S plasmid for B. subtilis RIK1285 expression system containing AmDyP gene. d) pBE-S plasmid for B. subtilis RIK1285 expression system containing AmLac, AmLac ∆SP, ScLac, ScLac ∆SP, SvLac, and SvLac ∆SP genes.

**Table 1: Primers used in this study. Highlighted sequences are complementary overhangs to backbones for Gibson Assembly.**

| **#** | **Primer name** | **Forward 5’-3’** | **Reverse 5’-3’** |
| --- | --- | --- | --- |
| 1 | Bs SP DyP2 pNZ | gaaaggaggtgaccaccatgATGAGCGATGAACAGAAAAAG | gcatgcctgcagtacccatgTTAGTGGTGATGGTGATGAT |
| 2 | DyP2 pNZ | gaaaggaggtgaccaccatgATGCCGGTTGATCTGAGCAC | gcatgcctgcagtacccatgTTAGTGGTGATGGTGATGATGC |
| 3 | SCLAC pNZ | gaaaggaggtgaccaccatgATGGATCGTCGCGGATTCAA | gcatgcctgcagtacccatgTTAGTGGTGATGGTGATGATG |
| 4 | SCLAC ∆SP pNZ | gaaaggaggtgaccaccatgATGCCAGCCGCTAAAGGAAT | gcatgcctgcagtacccatgTTAGTGGTGATGGTGATGATG |
| 5 | AMLAC pBE | gcaaaaggagagggacgcgtATGGCTAAAATGACACGCCG | aactgtgataaactaccgcaTTAGTGGTGATGGTGATGATG |
| 6 | AMLAC ∆SP pBE | gcaaaaggagagggacgcgtATGGGAACCCCGAGAAGAAT | aactgtgataaactaccgcaTTAGTGGTGATGGTGATGATG |
| 7 | SCLAC pBE | gcaaaaggagagggacgcgtATGGATCGTCGCGGATTCAA | aactgtgataaactaccgcaTTAGTGGTGATGGTGATGATG |
| 8 | SCLAC ∆SP pBE | gcaaaaggagagggacgcgtATGCCAGCCGCTAAAGGAAT | aactgtgataaactaccgcaTTAGTGGTGATGGTGATGATG |
| 9 | SVLAC pBE | gcaaaaggagagggacgcgtATGGATCGGCGGGGATTTAA | aactgtgataaactaccgcaTTAGTGGTGATGGTGATGATG |
| 10 | SVLAC ∆SP pBE | gcaaaaggagagggacgcgtATGGGGCCTGCTAAAACAGC | aactgtgataaactaccgcaTTAGTGGTGATGGTGATGATGC |
| 11 | Bs SP DyP2 pBE | gcaaaaggagagggacgcgtATGAGCGATGAACAGAAAAAG | aactgtgataaactaccgcaTTAGTGGTGATGGTGATGAT |
| 12 | DyP2 pBE | gcaaaaggagagggacgcgtATGCCGGTTGATCTGAGCAC | aactgtgataaactaccgcaTTAGTGGTGATGGTGATGATGC |
| 13 | SCLAC ∆SP Fus pBE | gcaaaaggagagggacgcgtATGCCAGCCGCTAAAGGAAT | GTGCTCAGATCAACCGGCATCGCTTGGAAGTACAGGTTTT |
| 14 | DyP2 Fus pBE | ATGCCGGTTGATCTGAGCAC | aactgtgataaactaccgcaTTAGTGGTGATGGTGATGATGC |
| 15 | SCLAC ∆SP Fus pBE | gcaaaaggagagggacgcgtATGCCAGCCGCTAAAGGAAT | agctcctcgcccttgctcacCGCTTGGAAGTACAGGTTTT |
| 16 | GFP Fus pBE | gtgagcaagggcgaggagct | aactgtgataaactaccgcaTTActtgtacagctcgtccatgc |
| 17 | pBE linearization | tgcggtagtttatcacagttaaattg | acgcgtccctctccttt |
| 18 | AMLAC Pvsi  pUC19 | tcgatcgaaggagagctgcaATGGCTAAAATGACACGCCG | TCCTCTAGAGTCGACctgcaTTAGTGGTGATGGTGATGATG |
| 19 | AMLAC ∆SP Pvsi pUC19 | TCGATCGAAGGAGAGctgcaATGGGAACCCCGAGAAGAAT | TCCTCTAGAGTCGACctgcaTTAGTGGTGATGGTGATGAT |
| 20 | SCLAC Pvsi pUC19 | TCGATCGAAGGAGAGctgcaATGGATCGTCGCGGATTCAA | TCCTCTAGAGTCGACctgcaTTAGTGGTGATGGTGATGATG |
| 21 | SCLAC ∆SP Pvsi pUC19 | TCGATCGAAGGAGAGctgcaATGCCAGCCGCTAAAGGAAT | TCCTCTAGAGTCGACctgcaTTAGTGGTGATGGTGATGATG |
| 22 | SVLAC Pvsi pUC19 | TCGATCGAAGGAGAGctgcaATGGACAGACGCGGCTTCAA | TCCTCTAGAGTCGACctgcaTTAGTGGTGATGGTGATGATGC |
| 23 | SVLAC ∆SP Pvsi pUC19 | TCGATCGAAGGAGAGctgcaATGGGGCCTGCTAAAACAGC | TCCTCTAGAGTCGACctgcaTTAGTGGTGATGGTGATGATGC |
| 24 | SCLAC SP pUC19 | TCGATCGAAGGAGAGctgcaATGGATCGTCGCGGATTCAATCG | GTGCTCAGATCAACCGGCATAGCCGCCCCAGCCACTTCTG |
| 25 | DyP2 pUC19 | ATGCCGGTTGATCTGAGCAC | TCCTCTAGAGTCGACctgcaTTAGTGGTGATGGTGATGATGC |
